# Determinants of hyperemesis gravidarum among pregnant women attending antenatal care at public and private hospitals in Bahir Dar City, North-West Ethiopia, 2022: a multicenter unmatched case control study

**DOI:** 10.1186/s12905-023-02386-0

**Published:** 2023-05-03

**Authors:** Lakachew Asrade, Daniel Misikir, Hiwotemariam Alemu, Amare Belachew, Hunegnaw Almaw

**Affiliations:** College of Medicine and Health Science, Bahir Dar University, Bahir Dar, Ethiopia

**Keywords:** Hyperemesis Gravidarum, Determinant, Factors, Ethiopia

## Abstract

**Introduction:**

Hyperemesis gravidarum is a severe form of nausea and vomiting during pregnancy characterized by more than 5% weight loss and ketonuria. Although there are cases in Ethiopia, there is still insufficient information regarding the determinant factors of hyperemesis gravidarum.This finding helps to decrease maternal as well as fetal complications of hyperemesis gravidarum by early identification of pregnant mothers who are at high risk. This study aimed to assess determinants of hyperemesis gravidarum among pregnant women attending antenatal care at public and private hospitals in Bahir Dar, North-West Ethiopia, 2022.

**Method:**

A multicenter, facility-based, unmatched case-control study was conducted on 444 pregnant women (148 cases and 296 controls) from January 1 to May 30. Women with a documented diagnosis of hyperemesis gravidarum on the patient chart were considered as cases, and women who attended antenatal care service without hyperemesis gravidarum were assigned as controls. Cases were selected using a consecutive sampling technique, whereas controls were selected using systematic random sampling technique. Data were collected using an interviewer-administered structured questionnaire. The data were entered into EPI-Data version 3 and exported into SPSS version 23 for analysis. Multivariable logistic regression was performed to identify determinants of hyperemesis gravidarum at a p-value of less than 0.05. An adjusted odds ratio with a 95% confidence interval was used to determine the direction of association.

**Results:**

Living in urban (AOR = 2.717, 95% CI : 1.693,4.502), primigravida (AOR = 6.185, 95% CI: 3.135, 12.202), first& second trimester of pregnancy (AOR = 9.301, 95% CI: 2.877,30.067) & (AOR = 4.785, 95% CI: 1.449,15.805) respectively, family history of hyperemesis gravidarum (AOR = 2.929, 95% CI: 1.268,6.765), helicobacter pylori (AOR = 4.881, 95% CI: 2.053, 11.606) & Depression (AOR = 2.195, 95% CI: 1.004,4.797) were found to be determinants of hyperemesis gravidarum.

**Conclusion:**

Living in an urban area, primigravida woman, being in the first and second trimester, having family history of hyperemesis gravidarum, Helicobacter pylori infection, and having depression were the determinants of hyperemesis gravidarum. Primigravid women, those living in urban areas, and women who have a family history of hyperemesis gravidarum should have psychological support and early treatment initiation if they develop nausea and vomiting during pregnancy. Routing screening for Helicobacter pylori infection and mental health care for a mother with depression at the time of preconception care may decreases hyperemesis gravidarum significantly during pregnancy.

## Introduction

Hyperemesis gravidarum (HEG) is defined as persistent nausea and vomiting during pregnancy not related to other causes with an objective measure of acute starvation that includes ketonuria on urine analysis and weight loss of at least 5% of pre-pregnancy weight or at least 3 kg if pre-pregnancy weight is not known. It may also be associated with electrolyte and acid-base abnormalities, which include hypochloremia, hypokalemia, and hyponatremia [1]. HEG is a severe form of morning sickness and is one of the most common pregnancy-related complications [2]. HEG is common in the first and second trimesters, peaks at gestational ages of 8–12 weeks, and usually subsides at 20 weeks. However, a small percentage of pregnant women may have symptoms until delivery [3]. The prevalence of HEG globally is approximately 0.5–4.8% [3]. In Finland, the prevalence of HEG is 1.3% [4] and in Ethiopia, the prevalence of HEG ranges from 4.4% [5] to 8.2% [6].

The consequences of HEG, if left untreated and not prevented early, are both maternal and fetal complications. Maternal complications include shock, electrolyte abnormalities like hypokalemia, nutritional deficiencies, psychological disease, gastrointestinal trauma, and neurological damage [7]. Sometimes it could be the reason for an elective termination of pregnancy [8]. Fetal complications occur more frequently in pregnant women with HEG who do not gain adequate weight of at least 7 kg. These include a smaller head circumference, higher rate of preterm labor, low five-minute Apgar score, and also a higher risk for depression, bipolar disorder, and anxiety during adulthood [9]. It is also one of the most common health problems in the community during pregnancy, and it affects daily activities of women’s lives; patients will lose time and money from paid employment or private work, posing a significant economic burden on the country at large [7]. HEG also has a negative impact on quality of life and daily functioning. It also affects physical, social, and emotional functioning, bodily pain, general health perception, and mental health [10].

By reviewing different literature, the determinants of HEG from socio-demographic factor include younger age, being unmarried, having low educational status, having low social support, and having lost employment. From obstetric and medical perspectives, factors including primigravida, nulliparity, multiple pregnancy, history of abortion, history of dysmenorrhea, self or family history of HEG, history of pre-pregnancy motion sickness, Helicobacter pylori infection, and history of depression are significantly associated with HEG [2, 3, 6–8, 10–17].Knowing the determinant factors of HEG will help to reduce maternal and fetal complications by providing active screening for pregnant women who are at risk, close follow-up, and initiation of early management. By doing this, the progression of HEG can be slowed down significantly [3]. Despite a lot of research done on HEG, mechanism of the disease is largely unknown, and the other thing regarding risk factors is still debated in the literature with conflicting results. Determinant factors of HEG are different in different areas. In Ethiopia, despite having cases of severe forms, there are limited studies, particularly no research done in the Amhara region.

## Method

### Study area

The study was conducted in public and private hospitals in Bahir Dar city. Bahir Dar is the capital city of the Amhara national regional state, located 565 km northwest of the capital city of Ethiopia, Addis Ababa, with an altitude of 1799 m above sea level and a warm and temperate climate. The total population of Bahir Dar city was 308,877, of which 157,527 (51%) were male and 151,350 (49%) were female. The study was conducted in three government hospitals that include Tibebe Gihon Specialized Hospital, Felege Hiwot Referral Hospital, and Addisalem Primary Hospital. This study was also conducted in four private hospitals, including Gamby Teaching Hospital, Afilas General Hospital, Addinas General Hospital, and Dream Care General Hospital, which serve more than ten million people from the Amhara and Benshangul regions.

### Study design and period

Multicenter, facility-based, unmatched case control study was conducted from January 1 to May 30, 2022, G.C., in public and private hospitals in Bahir Dar, North West, Ethiopia. **Population**.

#### Source population

##### Cases

pregnant women who were admitted for the diagnosis of HEG in the study hospitals.

##### Controls

pregnant women who visited antenatal care in the study hospitals without having HEG.

#### Study population

##### Cases

pregnant women who were admitted for the diagnosis of hyperemesis gravidarum in the study hospitals during the study period.

##### Controls

pregnant women who visited antenatal care in the study hospitals without hyperemesis gravidarum during the study period.

### Sample size estimation and sampling technique

Sample size was calculated by Epi Info software version 7 using the Fleiss continuity correction double proportion formula. By using 80% power, a 95% confidence interval, and a case-to-control ratio of 1:2, Based on a study conducted in Mekelle, Ethiopia, by taking nulliparous as a determinant factor, the odds ratio was 1.97, the percent of cases exposed was 36.2, and the percent of controls exposed was 22.4 ( 7). The maximum sample size obtained was 404 (135 cases and 269 controls), and by adding a 10% non-response rate, the final sample size was 444 (148 cases and 296 controls).

### Sampling technique and procedures

Cases were selected by a consecutive sampling technique. All cases that were diagnosed as HEG by physicians and documented on the chart were selected with daily monitoring of all new admissions until the sample size was fulfilled in the study hospitals. Controls were selected from an ANC unit with no documentation of HEG on the chart by systematic random sampling on the same day of HEG diagnosis. The data were collected by three trained midwives using an Amharic version of a questionnaire, supervised by two residents.

### Inclusion criteria

#### Cases

pregnant mothers who were diagnosed with HEG by a physician and were admitted to private and public hospitals.

#### Controls

pregnant mothers who visited the ANC without HEG and shouldn’t have nausea and vomiting due to other causes.

### Exclusion criteria

Critically ill pregnant mothers were excluded from the study. The reason is the difficulty of getting the necessary information.

### Operational definitions

#### Hyperemesis gravidarum

pregnant mothers who had frequent nausea and vomiting not related to other causes and who had acute starvation, ketonuria on urine analysis, and weight loss of at least 5% of their pre-pregnancy weight [1].

#### Cases

pregnant women who were diagnosed with hyperemesis gravidarum by the clinicians based on the clinical and laboratory parameters.

#### Controls

a pregnant mother who visited antenatal care without hyperemesis gravidarum and shouldn’t have nausea and vomiting due to other causes.

#### Ketonurea

+2 and above ketone value on urine dipstick test.

#### Critically ill

pregnant mothers whose Glasgow coma scale is less than 15 documented on the chart.

#### Depression

It was measured by using the Edinburgh Postnatal Depression Scale (EPDS), which is a 10-item short multiple-choice questionnaire important for measuring depression during pregnancy or after giving birth, and each question has four options scored with 0, 1, 2, or 3. The total score is found by adding together the scores for each of the 10 items. The validation study shows that mothers who scored 12 or above are suffering from depression [18].

**Social Support**: It was assessed by using the Oslo Social Support Scale (OSSS-3), which consists of a 3-item questionnaire. There are 3 categories of social support: scores of 3–8 have poor social support, scores 9–11 have moderate social support, and scores 12–14 have strong social support [19].

### Data collection

Data were collected from mothers using a face-to-face interview questionnaire and chart reviews for laboratory tests and ultrasound results.All the data collectors collected the data after obtaining written informed consent from all study participants; for those who were less than eighteen years old, written informed consent was obtained from both study participants and their families. The questionnaire was adapted from previous literature (3, 7, 8, 10, 13, 15 and 17). Originally, the questionnaire was prepared in English, translated to the local language (Amharic), and translated back to English by two independent persons to ensure its consistency.

### Data processing and analysis

Data were entered into Epi Data version 3 and then exported to SPSS version 23 software packages for analysis. However, the data were checked for completeness before being entered into Epi Info. Statistics such as the mean, percentage, and standard deviation were determined for both cases and controls. Determinant factors for HEG were cross-tabulated for cases and controls. Bi-variable and multivariable logistic regression was used for associations. Determinant factors that had association in the bi-variable logistic regression with the enter model (p-value < 0.2) were chosen for multivariable logistic regressions analysis. The variables with p < 0.05 in multivariable logistic regressions were considered statistically significant determinants for HEG. The adjusted odds ratio (AOR) with a 95% confidence interval (CI) was used to assess the strength of the association. Model fitness was checked with the Hosmer and Lemeshow goodness of fit test (with a p value of 0.881). Multicollinearity tests were done, and all determinants had a variance inflation factor less than 10, indicating that there was no high correlation between the independent variables.

## Results

### Maternal socio-demographic characteristics

In this study, a total of 148 cases and 296 controls were successfully interviewed, resulting in a response rate of 100%. 57 (38.5%) of the cases and 116 (39.2%) of the controls were in the age range of 25 to 29 years. The mean age of cases and controls was 26.7 and 28.5, respectively. About three-fourths of the cases and 155 (52.4%) of the controls were living in urban areas, and 134 (90.5%) of the cases and 275 (92.9%) of the controls were married.From the participants, 127 (85.5%) of cases and 257 (86.2%) of controls were Orthodox. About 66 (44.6%) of cases and 92 (31.1%) of controls had college-level or higher educational status, whereas 26 (17.6%) of cases and 107 (36.1%) of controls didn’t attend formal educations. About 48 (32.4%) cases and 82 (27.3%) controls were employed. Moreover, 43 of the cases (29.1%) and 118 of the controls (39.9%) were housewives. Based on social support, 58 (39.2%) of cases and 152 (51.4%) of controls had moderate social support, whereas 16.2% of cases and 7.8% of controls had poor social support. (Table 1).

**Table 1:** Socio-demographic characteristics of respondents among pregnant women who visit antenatal care at public & private hospitals in Bahir Dar city, North-West Ethiopia, 2022 (N = 444)

Characteristics	Category	Cases		Controls	
		N	%	N	%
Age	15–19	4	2.7	18	6.1
	20–24	30	20.3	85	28.7
	25–29	57	38.5	116	39.2
	30–34	42	28.4	56	18.9
	35–39	11	7.4	18	6.1
	40–44	3	2.7	3	1
Residency	Urban	111	75	155	52.4
	Rural	37	25	141	47.6
Marital status	Married	134	90.5	275	92.9
	Unmarried	14	9.5	21	7.1
Religion	Orthodox	127	85.5	257	86.8
	Muslim	10	6.8	25	8.4
	Protestant	7	4.7	8	2.7
	Catholic	4	2.7	6	2
Educational status	No formal education	26	17.6	107	36.1
	Primary school	31	20.9	49	16.6
	Secondary school	25	16.9	48	16.2
	College & above	66	44.6	92	31.1
Occupation	Employed	48	32.4	82	27.7
	Merchant	27	18.2	38	12.8
	House Wife	43	29.1	118	39.9
	Student	6	4.1	9	3
	Farmer	24	16.2	49	16.6
Oslo social support scale(OSSS-3)	Poor social support	24	16.2	23	7.8
	Moderate social support	58	39.2	152	51.4
	Strong social support	66	44.6	121	40.9

### Obstetric and gynecologic, medical and psychiatric factors characteristics

Out of the total number of women interviewed, most of those with HEG (73.6%) and without HEG (93.6%) were multigravida, while one-fourth of cases were primigravida. Nearly 40% of cases and more than half (55.7%) of controls were multiparas. About 45 (40.9%) of cases and 12 (4.3%) of controls had a history of HEG. From pregnant mothers, 42 (38.5%) of cases and 19 (6.9%) of controls had a history of abortion. Most of the pregnancies, 141 (95.3%) among cases and 289 (97.6%) among controls, were singletons. The mean gestational age of cases and controls was 11.1 ± 2.8 and 21.5 ± 2.6 weeks, respectively. About two-thirds of cases (64.9%) were admitted during the first trimester and one-third (32.4%) of HEG cases were admitted during the second trimester, and 23 (2.7%) were admitted in the third trimester. Around one-fourth (25.7%) of the cases and 20 (6.8%) of the controls reported a history of HEG in their mothers and sisters. Most of the cases (90.4%) and controls (92.2%) reported that the pregnancy was planned. About one-fourth (25%) of HEG mothers and 21.3% of controls had a history of dysmenorrhea. Concerning medical characteristics, a history of pre-pregnancy motion sickness was reported by 28 (18.9%) of cases and 42 (14.2%) of controls. About 28 (18.9%) of the cases and 5.7 (17%) of the controls were seropositive for Helicobacter pylori (H. pylori) infection. Regarding history of depression, about 21 (14.2%) patients were having depression, whereas only 21 (7.1%) of controls had depression (Table 2).

**Table 2:** Obstetric and Gynecologic, Medical and Psychiatric characteristics of respondents among pregnant women who visit antenatal care at public & private hospitals in Bahir Dar city, North-West Ethiopia, 2022 (N = 444)

Characteristics	Category	Case		Control	
		N	%	N	%
Gravidity	Primigravida	39	26.4	19	6.4
	Multigravida	109	73.6	277	93.6
Parity	Primipara	41	27.7	111	37.5
	Multipara	101	40.5	165	55.7
Previous history of HEG	Yes	45	40.9	12	4.3
	No	65	59.1	264	95.7
Previous history of abortion	Yes	42	38.5	19	6.9
	No	67	61.5	258	93.1
Types of gestation	Singleton	141	95.3	289	97.6
	Multiple	7	4.7	7	2.4
GA in weeks	First trimester	96	64.9	121	40.9
	s trimester	48	32.4	134	44.3
	Third trimester	4	2.7	84	14.9
Family history of HEG	Yes	38	25.7	20	6.8
	No	110	74.3	276	93.2
Pregnancy status	Planned	134	90.5	273	92.2
	Unplanned	14	9.5	23	7.8
Dysmenorrhea history	Yes	37	25	63	21.3
	No	111	75	233	78.7
History of pre-pregnancy motion	Yes	28	18.9%	42	14.2%
	No	120	81.1%	254	85.8%
Helicobacter Pylori serostatus	Positive	28	18.9%	17	5.7%
	Negative	120	81.1%	279	94.3%
Depression	Yes	21	14.2	21	7.1
	No	127	85.8	275	92.9

### Determinant factors of hyperemesis gravidarum

First, variables were tested using bivariable analysis between independent variables and HEG. These analyses revealed that residency, social support, gravidity, types of gestation, gestational age, having a family history of HEG, helicobacter pylori serostatus, and depression were associated with the development of HEG at a p-value of < 0.2 were entered into multi-variable logistic regression analyses. After controlling the possible confounders: being urban, having primigravity, being in the first and second trimesters of pregnancy, having a family history of HEG, having a Helicobacter pylori infection and having a depression history were the determinants of hyperemesis gravidarum in multi-variable logistic regression analysis at a p-value of < 0.05. The direction of association was computed by using an adjusted odds ratio with a 95% confidence interval.

The result showed that pregnant women from urban areas were significantly associated with HEG, with an odds ratio of 2.717 times more likely to develop HEG as compared to pregnant women from rural areas (AOR = 2.717, 95% CI: 1.639, 4.502). In the same manner, mothers with primigravity had a 6.185 times higher odds ratio to have HEG than multigravida mothers (AOR = 6.185, 95% CI: 3.135, 12.202). Pregnant mothers in the first trimester and second trimester of pregnancy had 9.301 and 4.785 higher odds ratios to develop HEG compared to pregnant mothers who were in the third trimester, respectively (AOR = 9.301, 95% CI: 2.877, 30.067) and (AOR = 4.785, 95% CI: 1.449, 15.805). Similarly, pregnant women with a family history of hyperemesis gravidarum were 5.020 times more likely to have HEG as compared to those who had no family history of HEG (AOR = 5.020, 95% CI = 2.599, 9.697).

Having helicobacter infection was significantly associated with hyperemesis gravidarum, with a higher odds ratio of 4.369 as compared with pregnant women without helicobacter infections (AOR = 4.369, 95% CI: 2.014, 9.480). Similarly, having a history of depression was associated with a 2.195 higher odds ratio to develop hyperemesis gravidarum compared with pregnant mothers without depression (AOR = 2.195, 95% CI: 1.004, 4.797) (Table 3).

**Table 3:** Determinants of hyperemesis gravidarum among pregnant women who visit antenatal care at public & private hospitals in Bahir Dar city, North-West Ethiopia, 2022 (N = 444)

Characteristics	Category	Case N (%)	Control N (%)	COR(95% CI)	AOR(95% CI)	P-value
Residency	Urban	111(75)	155(52.4)	2.729(1.764,4.222)	2.717(1.639,4.502)**	< 0.001
	Rural	37(25)	141(47.6)	1	1	
Oslo social support scale(OSSS-3)	Poor social support	24(16.2)	23(7.8)	1.913(1.003,3.649)	2.008(0.935,4.312)	0.074
	Moderate social support	58(39.2)	152(51.4)	0.700(0.457,1.071)	0.754(0.453,1.254)	0.276
	Strong social support	66(44.6)	121(40.8)	1	1	
Gravidity	Primigravida	39(26.4)	19(6.4)	5.216(2.887,9.424)	6.185(3.135,12.202)**	< 0.001
	Multigravida	109(73.6)	277(93.6)	1	1	
Types of gestation	Singleton	141(95.3)	289(97.6 )	1	1	
	Multiple	7(4.7)	7(2.4)	2.050(0.705,5.958)	1.576(0.452,5.496)	0.475
GA in weeks	1st trimester	96(64.9)	121(40.9)	8.727(3.030,25.141)	9.301(2.877,30.067)**	< 0.001
	2nd trimester	48(32.4)	134(44.3)	4.0331(1.375,11.817)	4.785(1.449,15.805)*	0.010
	3rd trimester	4(2.7)	84(14.9)	1	1	
Family history of HEG	Yes	38(25.7)	20(6.8)	1.411(0.835,2.386)	5.020(2.599,9.697)**	< 0.001
	No	110(74.3)	276(93.2)	1	1	
History of pre-pregnancy motion sickness	Yes	28(18.9)	42(14.2)	2.387(1.418,4.018)	1.706(0.893,3.262)	0.106
	No	120(81.1)	254(85.8)	1	1	
Helicobacter Pylori serostatus	Positive	28(18.9)	17(5.7)	3.829(2.020,7.259)	4.369(2.014,9.480)**	< 0.001
	Negative	120(81.1)	279(94.3)	1	1	
Depression	Yes	21(14.2)	21(7.1)	2.165(1.141,4.108)	2.195(1.004,4.797)*	0.049
	No	127(85.8)	275(92.9)	1	1	

* = p-value < 0.05; ** = p-value < 0.001; 1 = Reference; AOR = Adjusted Odd Ratio, COR = crude odds ratio, CI = Confidence interval

## Discussion

This study identified determinant factors that have been associated with HEG in Bahir Dar, Ethiopia. Identification of determinant factors could reduce adverse perinatal outcomes, hospitalization, time lost from paid employment, and emotional and psychological problems. This study found that pregnant women who lived in urban areas were significantly more likely to have HEG than pregnant women who lived in rural areas. This finding is similar to that of a study done at Bale Zone, south Ethiopia [8]. The possible explanation for this association could be a difference in triggering factors. Living in an urban environment might have triggering factors for HEG, such as smelling from poor waste disposal systems. Another explanation could be that most pregnant mothers from urban areas are overweight and obese, which will contribute to the development of HEG. In addition, urban women might be psychologically more sensitive, which may contribute to acid secretion in the stomach. This finding contradicts a study done in Turkey, where socio-demographic parameters showed no significant difference between the case and control groups [2]. The discrepancy could be explained by the different living standards between rural and urban areas compared with our setup, which has a difference in housing conditions, environmental sanitation, sewerage systems, and ventilation between urban parts of Turkey and Ethiopia.

Pregnant women with primigravida were significantly associated with hyperemesis gravidarum. This finding is consistent with a study done in Addis Ababa, Ethiopia [6], Egypt [20], Finland [4] and England [12]. This may be due to a stressful condition occurring because of no previous experience, and exposure to high levels of HCG for the first time may increase the likelihood of hyperemesis gravidarum. However, this contradicts a study done in Nigeria, in which multiparity was a risk factor. The possible explanation given in this study is that most women in the study included were above the age of 30; in this age group, women might have had two or more deliveries because of the cultural practice of early marriage and childbearing in Nigeria [21].

Pregnant mothers in the first and second trimesters were at higher risk of developing HEG compared to pregnant mothers who were in the third trimester. This finding is consistent with a study done in Jimma (Southwest Ethiopia), Bale Zone (South Ethiopia), Mekelle City (North Ethiopia), and the University of Michigan [7, 8, 22, 23]. This could be explained by the body’s reaction to the pregnancy hormone, especially human chorionic gonadotropin, which is produced in higher amounts in the first and second trimesters than in the third trimester. The other explanation could be woman’s subconscious mind’s attempt to reject pregnancy in the early trimester that is adapted later in the third trimester.

A family history of HEG in this study had a strong association with the development of HEG. This finding is consistent with a study done in North Ethiopia that found a significant association with HEG [12]. Similarly, a review article by Gabra A in 2018 found that there was a strong association between family history and HEG [1]. This finding is also similar to a study done in Uganda, Nigeria, and the USA [3, 21, 24], which shows a significantly higher risk of hyperemesis in women whose sister’s or mother’s had hyperemesis gravidarum. This could be because of the familial aggregation gene, mainly the growth differentiation factor 15 gene and its action in the chemoreceptor trigger zone of the brain which has genetic associations with HEG. However, this is contradicted in a study done in Bale Zone, south Ethiopia, in which no association was found [7]. The possible reason for this contradictory result could be that the family history of hyperemesis gravidarum was based on self-report, and the patient might deny having a family history of HEG.

In this study, having a helicobacter pylori infection was also found to have a significant association with HEG. This finding is consistent with a study done in Addis Ababa (Ethiopia), North Ethiopia, Egypt and Iraq [7, 20, 22, 25]. A meta-analysis study done in 2015 showed that it is an important risk factor, especially in developing countries [26]. The possible explanation for this could be that H. pylori infection may aggravate the hormone-induced changes in the chemoreceptor trigger zone in the brain stem, including the vomiting center and electric functioning of the stomach, which could lead an infected pregnant woman to develop severe nausea and vomiting [27].

This study showed that having depression was significantly associated with hyperemesis gravidarum. Similarly, a study done in Turkey showed that depression is significantly associated with nausea and vomiting in the early trimester of pregnancy [2]. A study done in Norwegian also showed that depression was associated with a higher odds ratio for hyperemesis gravidarum [28]. It may be due to inadequate food intake, loss of energy, poor socialization, no future, and loss of hope as a result of depression, which will increase nausea and vomiting during pregnancy, as supported by the psychosocial theory of hyperemesis gravidarum.

## Conclusion

This study concludes that being from an urban area, being primigravida, being in the first and second trimesters of pregnancy, family history of hyperemesis gravidarum, helicobacter pylori infection and depression were the determinants of hyperemesis gravidarum. Pregnant women who come from urban areas, primigravida women, and those with a family history of HEG should visit a health facility for early treatment of symptoms like nausea and vomiting that will decrease progression to HEG. Pregnant women in the first trimester were significantly affected by HEG. Therefore, health care providers should take HEG into account at the first ANC visit. Routing screening for H. pylori infection at the time of preconception counseling may decrease hyperemesis gravidarum significantly during pregnancy. Depression must be treated during the preconceptional counseling period, and extra psychological support may be necessary during treatment and follow-up.

## Data Availability

The data of this study cannot be shared publically. The reason is the presence of confidential issues of the study participant’s information. The data are available from corresponding author and can access with request.
